# The Critique

**Published:** 1898-04

**Authors:** 


The Critique, a journal devoted to Homœopathy and published in Denver, is organizing a wonderful outing tour through the Rocky Mountains next summer, in connection with the American Institute of Homœopathy. This wonderful excursion will cost only sixty dollars ($60.00). Those who wish to enjoy this excursion should subscribe *now*. For circulars and full information address Dr. J. Wylie Anderson, 16 Steele Block, Denver, Colorado.

